# Cloning, expression and characterization of a β-d-xylosidase from *Lactobacillus rossiae* DSM 15814^T^

**DOI:** 10.1186/s12934-016-0473-z

**Published:** 2016-05-03

**Authors:** Erica Pontonio, Jennifer Mahony, Raffaella Di Cagno, Mary O’Connell Motherway, Gabriele Andrea Lugli, Amy O’Callaghan, Maria De Angelis, Marco Ventura, Marco Gobbetti, Douwe van Sinderen

**Affiliations:** Department of Soil, Plant and Food Science, University of Bari Aldo Moro, Via G. Amendola 165/A, 70126 Bari, Italy; School of Microbiology, University College Cork, Cork, Ireland; Alimentary Pharmabiotic Centre, University College Cork, Cork, Ireland; Laboratory of Probiogenomics, Department of Life Sciences, University of Parma, Parma, Italy

**Keywords:** Xylo-oligosaccharides, Sourdough, Prebiotic, Gut microbiota, Functional foods, Probiotic

## Abstract

**Background:**

Among the oligosaccharides that may positively affect the gut microbiota, xylo-oligosaccharides (XOS) and arabinoxylan oligosaccharides (AXOS) possess promising functional properties. Ingestion of XOS has been reported to contribute to anti-oxidant, anti-bacterial, immune-modulatory and anti-diabetic activities. Because of the structural complexity and chemical heterogeneity, complete degradation of xylan-containing plant polymers requires the synergistic activity of several enzymes. Endo-xylanases and β-d-xylosidases, collectively termed xylanases, represent the two key enzymes responsible for the sequential hydrolysis of xylan. Xylanase cocktails are used on an industrial scale for biotechnological purposes. *Lactobacillus rossiae* DSM 15814^T^ can utilize an extensive set of carbon sources, an ability that is likely to contribute to its adaptive ability. In this study, the capacity of this strain to utilize XOS, xylan, d-xylose and l-arabinose was investigated.

**Results:**

Genomic and transcriptomic analyses revealed the presence of two gene clusters, designated *xyl* and *ara*, encoding proteins predicted to be responsible for XOS uptake and hydrolysis and d-xylose utilization, and l-arabinose metabolism, respectively. The deduced amino acid sequence of one of the genes of the *xyl* gene cluster, LROS_1108 (designated here as *xylA*), shows high similarity to (predicted) β-d-xylosidases encoded by various lactic acid bacteria, and belongs to glycosyl hydrolase family 43. Heterologously expressed XylA was shown to completely hydrolyse XOS to xylose and showed optimal activity at pH 6.0 and 40 °C. Furthermore, β-d-xylosidase activity of *L. rossiae* DSM 15814^T^ was also measured under sourdough conditions.

**Conclusions:**

This study highlights the ability of *L. rossiae* DSM 15814^T^ to utilize XOS, which is a very useful trait when selecting starters with specific metabolic performances for sourdough fermentation or as probiotics.

**Electronic supplementary material:**

The online version of this article (doi:10.1186/s12934-016-0473-z) contains supplementary material, which is available to authorized users.

## Background

In recent years the use of prebiotics, in particular oligosaccharides, to modulate the gut microbiota composition and associated metabolic activities, together aimed at improving gut health, has enjoyed considerable scientific and commercial interest [1, 2]. Colonic fermentation of particular oligosaccharides into short chain fatty acids (SCFA) is believed to increase the number and metabolic activity of certain beneficial bacterial populations [1, 3]. Among the oligosaccharides that may positively alter the composition of the gut microbiota, xylo-oligosaccharides (XOS) and arabinoxylan oligosaccharides (AXOS) possess promising functional properties as they can be specifically fermented by intestinal commensals such as bifidobacteria and lactobacilli [4–6]. XOS resist hydrolysis by enzymes and/or the low pH present in human saliva, gastric and pancreatic juices, and are not absorbed during transit through the small intestine, thus reaching the colon where they are available as fermentable substrates for certain members of the resident microbiota [7]. It has previously been reported that ingestion of XOS contributes to anti-oxidant, anti-bacterial, immune-modulatory and anti-diabetic activities [8, 9]. Arabinoxylans represent 50 % of dietary fibers present in wheat bran [10, 11]. In particular, the highly polymerized arabinoxylan from bran may be hydrolyzed by specific bacteria possessing arabinoxylan-degrading enzymes [6].

XOS are oligomers that consist of 2–10 xylose residues connected through β-(1-4)-linkages. Their liberation is the result of (partial) hydrolysis of xylan, the major component of plant hemicelluloses. Because of the structural complexity (such as the presence of various side chains, in particular arabinose) and chemical heterogeneity, complete degradation of xylan-containing plant polymers requires the synergistic activity of several enzymes [12], such as endo-xylanase (endo-1,4-β-xylanase, E.C. 3.2.1.8), β-d-xylosidase (xylan-1,4-β-d-xylosidase, E.C. 3.2.1.37), α-glucuronidase (α-glucosiduronase, E.C.3.2.1.139), α-arabinofuranosidase (α-l-arabinofuranosidase, E.C. 3.2.1.55) and acetylxylan esterase (E.C. 3.1.1.72). Endo-xylanases and β-d-xylosidases (collectively named as xylanases) represent the two key enzymes responsible for the sequential hydrolysis of xylan. Endo-xylanases act on the homo-polymeric backbone of the β-1,4-linked xylan liberating xylo-oligomers [13], whereas β-d-xylosidases are active on these latter oligomers releasing xylose [14]. For the degradation of arabinoxylan, α-l-arabinofuranosidases are also needed because they cleave arabinose from the backbone and act in synergy with endoxylanases [15]. Xylanase cocktails are used on an industrial scale for de-inking of recycled paper [16], processing of wood pulp [17], improving bread dough baking and nutritional quality [18], hydrolysis of bitter molecules and liberation of aroma compounds during grape juice extraction and wine making [12].

In nature, a variety of microorganisms, including bacteria and fungi, encode xylanases and β-xylosidases [14]. These enzymes are either cell wall-associated, secreted into the extracellular environment or present in the cytoplasm. Based on amino acid sequence similarities xylanases and β-xylosidases are classified into glycoside hydrolase (GH) families 1, 3, 30, 39, 43, 51, 52, 54, 116 or 120. In particular, β-xylosidases belonging to the GH43 family, which are predominantly encoded by bacteria, hydrolyze the non-reducing ends of the xylo-oligomers using an inverting mechanism [14]. Despite the recognized potential of XOS as a prebiotic to target beneficial components of the human gut microbiota, in particular lactobacilli and bifidobacteria, very little is known about the enzymes used by the former group of bacteria to hydrolyze these complex substrates. To the best of our knowledge, β-d-xylosidase from *Lactobacillus brevis* is the only report on the characterization of such an enzyme in the *Lactobacillus* genus [9, 19].

Recently, the genomic annotation and comparative analysis of *L. rossiae* DSM 15814^T^ revealed the predicted presence of a number of extracellular or cell wall-associated polysaccharide-degrading enzymes, represented by putative cyclomaltodextrinase (E.C. 3.2.1.54; LROS_1707), α-amylase (E.C. 3.2.1.10; LROS_1584), β-glucosidase (E.C. 3.2.1.21; LROS_2047), mannosyl-glycoprotein endo-β-N-acetylglucosaminidase (E.C. 3.2.1.96; LROS_0612) and neopullulanase (EC 3.2.1.135; LROS_1707) enzymes [20]. Furthermore, genes involved in the degradation of arabinose and xylose-containing poly- and/or oligo-saccharides were predicted. *L. rossiae* is an obligatory hetero-fermentative lactic acid bacterium, which has been isolated from the gastrointestinal tract of humans [21] and animals [22], wheat sourdoughs [23–25], legumes [26], spelt flour [27] and pineapple [28]. *L. rossiae* was found to be a promising probiotic candidate thanks to its ability to survive under simulated gastric and intestinal conditions, and to stimulate immune-mediators by peripheral blood mononuclear cells [29]. Exposure to gastric and intestinal fluids is the main environmental stress that decreases viability of ingested probiotics [30, 31]. More in depth knowledge on XOS metabolism by *L. rossiae* is important from a biotechnological perspective to facilitate the selection of strains with specific metabolic performances to be used as starters for sourdough bread making, aimed at improving rheology and nutritional properties, or as probiotic for human applications.

In the current study we used a transcriptome approach to identify *L. rossiae* DSM 15814^T^ genes that were upregulated when this strain was grown on XOS-, xylose- or arabinose. One of the identified genes, *xylA*, was selected for further characterization and was cloned in *Lc. lactis* subsp. *cremoris* NZ9000 and the encoded recombinant enzyme was purified and characterized.

## Results

### Growth on XOS, xylan, d-xylose or l-arabinose

When maltose was used as a sole carbon and energy source in growth medium (see “Methods” section), *L. rossiae* was shown to increase its viable count from ca. 7.4 ± 0.1 to 9.4 ± 0.3 log CFU ml^−1^. The stationary phase of growth was reached after approximately 10 h, with a lag phase and μmax of 2.9 ± 0.2 h and 0.52 ± 0.03 log CFU ml^−1^ h^−1^, respectively. In the presence XOS, d-xylose or l-arabinose, *L. rossiae* was shown to exhibit similar growth kinetics. The increase of cell viability ranged from 7.3 ± 0.1 to 9.5 ± 0.3 log CFU ml^−1^, the values of λ varied from 2.7 ± 0.3 (l-arabinose) to 2.5 ± 0.1 h (XOS), and those of μmax from 0.27 ± 0.02 (l-arabinose and XOS) to 0.31 ± 0.03 Log CFU ml^−1^ h^−1^ (d-xylose). *L. rossiae* was not shown to exhibit appreciable growth in the presence of xylan, rye arabinoxylan, wheat arabinoxylan, arabinan, arabinogalactan and xyloglucan, as the sole carbon source (data not shown).

### Genome response of *L. rossiae* DSM 15814^T^ to growth on XOS

**Fig. 1:**
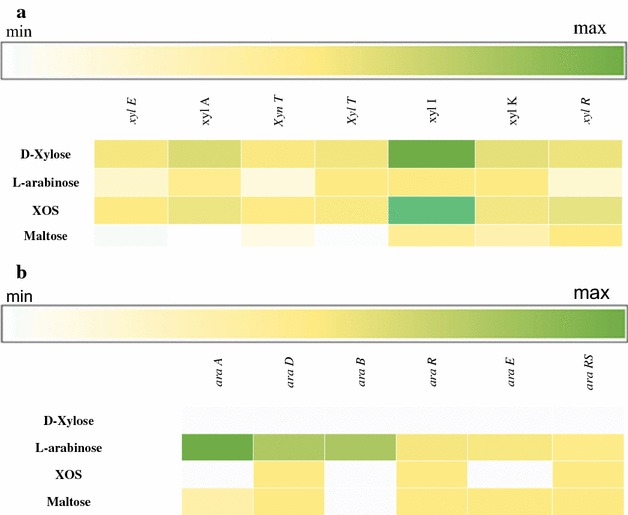
Heatmap based on the transcriptome analysis of *Lactobacillus rossiae* DSM15814^T^ grown on d-xylose, l-arabinose, XOS and maltose as the sole carbon source. XOS hydrolysis and utilization of the end product d-xylose (**a**) and the utilization of the l-arabinose (**b**) operon. The gene expression is expressed as RPKM (Reads per kilobase per million)

In order to investigate which genes are expressed when *L. rossiae* DSM 15814^T^ is grown in presence of XOS, d-xylose, l-arabinose or maltose (as a reference) as the sole carbon source, global gene expression was determined by RNAseq analysis. Compared to growth on maltose as the sole carbon source, various adjacent genes (designated *xylE*, *xylA*, *xynT*, *xylT*, *xylI*, *xylK* and *xylR*; and here referred to as the *xyl* cluster) were shown to exhibit transcriptional increases that ranged from 8.6 to 250 fold, or from 11.4 to 259.3 fold, when the strain was grown on XOS (Fig. 1a) or d-xylose, respectively. Furthermore, the co-located *araA*, *araD*, *araB*, *araR* and *araRS* genes, which encompass the *ara* gene cluster, predicted to encode enzymes for l-arabinose utilization, exhibited an increase in their transcription from 0.9 to 156 fold when l-arabinose was used as the only carbon source (Fig. 1b) (see below for details on putative functions).

### Genetic organization of the *ara* and *xyl* custers, and comparative analysis of the *xylA*-encoded β-d-xylosidase

**Fig. 2:**
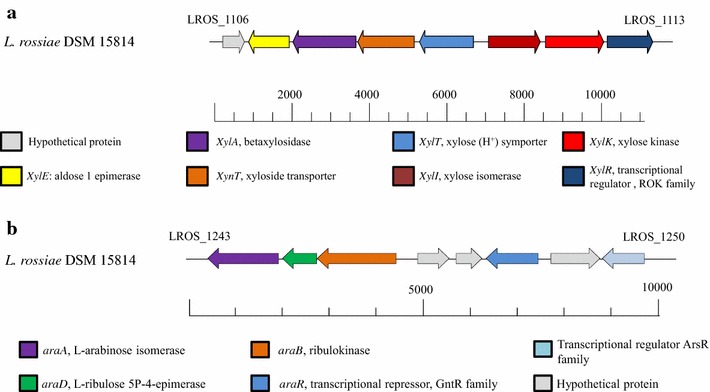
Schematic representation of the genetic organization (ca. 11 kb region) in *Lactobacillus rossiae* DSM 15814^T^. XOS hydrolysis and utilization of the end product d-xylose (**a**) and the utilization of the l-arabinose (**b**) operon. The size and orientation of each of the genes were deduced from their DNA sequences. The map was derived by the use of CloneManager Professional software (Scientific and Educational Software; USA)

**Fig. 3:**
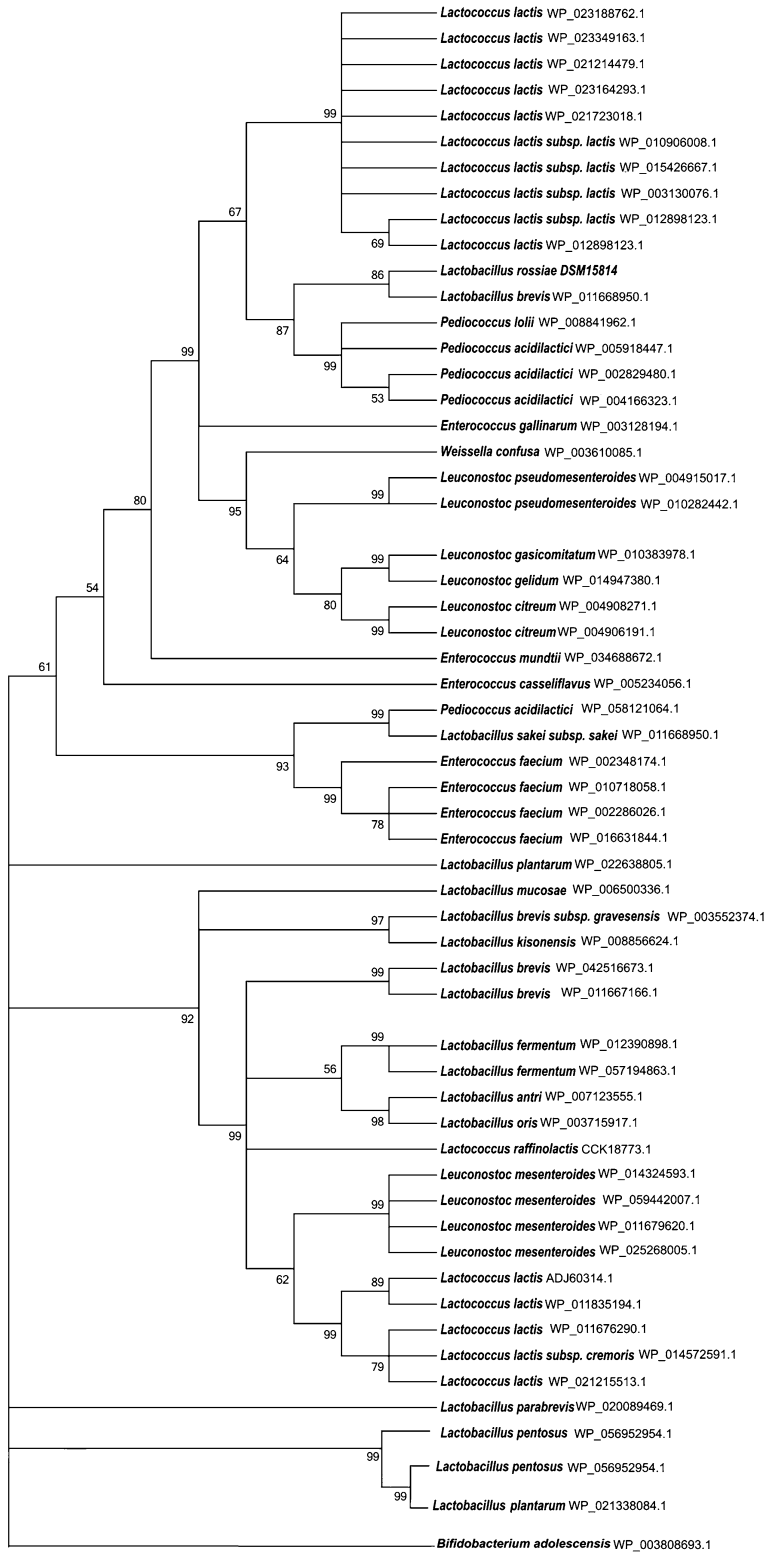
Phylogenetic tree showing the relationship between the amino acid sequences of β-d-xylosidase from *Lactobacillus rossiae* DSM15814^T^ and reference of sequences of some lactic acid bacteria in GenBank. The tree was constructed using the neighbour-joining software, numbers at the node are the bootstrap values (%). GroEL of *Bifidobacterium adolescentis* was used as outlier

As mentioned above, transcriptome analysis of *L. rossiae* DSM 15814^T^ revealed the presence of two gene clusters, *xyl* and *ara*, that are linked to XOS/d-xylose and l-arabinose metabolism, respectively (Fig. 2). The *xyl* cluster encompasses seven genes, which are schematically outlined in Fig. 2a, and which are predicted (based on BLAST-mediated similarity searches, Additional file 1: Table S1) to encode a xylose isomerase (E.C. 5.3.1.5; LROS_1111, designated here as *xylI*), a xylulose kinase (E.C 2.7.1.17; LROS_1112, designated here as *xylK*) and a ROK family transcriptional regulator (LROS_1113, designated here as *xylR*). Upstream of, but divergently oriented from the three genes mentioned above are genes encoding a predicted aldose 1-epimerase (E.C. 5.1.3.3; LROS_1107, designated here as *xylE*) and a putative β-d-xylosidase (EC3.2.1.37; LROS_1108, designated as *xylA*), followed by *xynT* (a putative xyloside transporter-encoding gene; LROS_1109) and *xylT* (predicted to specify a d-xylose proton symporter; LROS_1110). Thus, the genes in this *xyl* cluster represent proteins that may be involved in XOS metabolism (Kyoto Encyclopedia of Genes and Genomes database; Fig. 2). Among the genes of the *xyl* cluster, *xyl*A may have a key role in XOS hydrolysis as its encoded protein product is predicted to convert XOS into d-xylose. Alignment of the *L. rossiae* DSM 15814^T^*xylA*-encoded β-d-xylosidase with similar protein sequences from other lactic acid bacteria (NCBI website) produced the phylogenetic tree displayed in Fig. 3. This analysis shows that the β-d-xylosidase from *L. rossiae* DSM 15814^T^ belongs to a cluster consisting of mostly predicted β-d-xylosidases encoded by *Leuconostoc*, *Weissella* and *Pediococcus* species, and *Lc. lactis*.

The *ara* cluster is predicted to encompasses genes required for L-arabinose metabolism (Fig. 2b). In particular, based on BLAST similarity searches (Additional file 1: Table S1), it is predicetd that LROS_1243 encodes an l-arabinose isomerase (*araA*; E.C. 5.3.1.4), LROS_1244 specifies an l-ribulose 5P-4-epimerase (*araD*; E.C. 5.1.3.4), LROS_1245 encodes a ribulose kinase (*araB*; E.C 2.7.1.16), LROS_1248 a transcriptional repressor GntR family (*araR*;), LROS_1249 an aldose-1-epimerase (*araE*; E.C. 5.1.3.3) and LROS_1250 a transcriptional regulator 2C ArsR family (*araRS*). The complete conversion of l-arabinose into d-xylulose-5P is allowed by the sequential activities of AraA, AraB and AraD, respectively. Divergently orientated are three hypothetical genes with unknown function (LROS_1246, LROS_1247 and LROS_1249).

### XylA expression in *Lc. lactis* subsp. *cremoris* NZ9000 and characterization of β-d-xylosidase activity

**Fig. 4:**
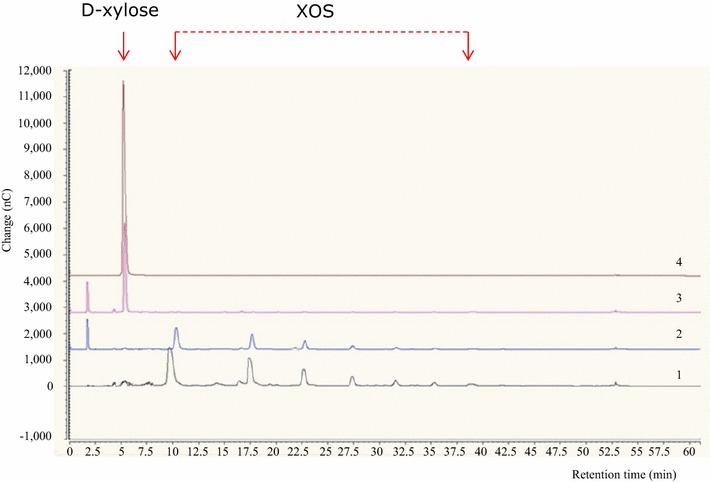
Hydrolysis of XOS as assessed by high-performance anion-exchange chromatography (HPAEC). *Lane 1*, 5 mg ml^−1^ (wt/vol) standard of XOS; *lane 2*, *Lactococcus lactis* subsp. *cremoris* NZ9000 containing the empty plasmid (pNZ8048) (negative control); *lane 3*; *Lc. lactis* subsp. *cremoris* NZ9000 containing the pNZ8048.1108 construct;* lane 4, 5* mg ml^−1^ (wt/vol) standard of d-xylose. Details on recombinant *Lc. lactis* subsp. *cremoris* NZ9000 are reported in “Methods” section

To demonstrate if *xylA* encodes, as predicted, a β-d-xylosidase capable of XOS hydrolysis, this gene was cloned into the protein expression vector pNZ8048, and placed under the transcriptional control of the inducible P_*nisA*_ promoter (see “Methods” section). In order to verify the hydrolytic activity of XylA, purified protein and crude cell extract (CCE) from Nisaplin-induced *Lc. lactis* subsp. *cremoris*, harbouring the pNZ8048-*xylA* were individually incubated for 24 h. Following incubation, CCE from *Lc. lactis*, harbouring the pNZ8048-*xylA*, showed hydrolytic activity towards XOS (Fig. 4). As expected, using identical experimental conditions, CCE from *Lc. lactis*, harbouring the empty pNZ8048 (negative control) did not exhibit measurable activity. In agreement with the comparative genome sequence analysis of *L. rossiae*, these results demonstrate that *xylA* specifies a β-d-xylosidase responsible for the observed XOS-degrading activity. Unfortunately, for unknown reasons the XylA protein completely lost its hydrolytic activity upon purification from the CCE (results not shown), and therefore further characterization of this enzyme was performed using XylA-containing CCE.

The effect of the pH on the activity of the recombinant His-tagged XylA was determined in Na-acetate (pH 3.0–6.0), phosphate (pH 6.0–7.0) and Tris–HCl (pH 7.0–9.0) buffer (Additional file 1: Figure S1). The enzyme was highly active (>80 %) in the range of pH of 5.0–7.0, displaying an optimum at pH 5.7. Above pH 7.0, enzyme activity rapidly decreased, being completely lost at pH 9.0. XylA enzyme activity was optimal at 40 °C, and it decreased thereafter (Additional file 1: Figure S1).

Using *L. rossiae* DSM 15814^T^ cells, enzyme activity was also determined under conditions that mimicked sourdough. After 24 h of fermentation, the xylosidase activity measured in sourdough was 4.4 ± 0.1 U, corresponding to 70 % of the maximum activity found in phosphate buffer. Following growth of *L. rossiae* DSM 15814^T^ in wheat flour hydrolysate medium (WFH), the CCE was shown to exhibit an activity of 5.6 ± 0.2 U.

## Discussion

The relatively large genome (genome size ~ 2.8 Mb) of *L. rossiae* DSM 15814^T^ is believed to reflect the metabolic and adaptive versatility of the species, mirroring an impressive potential to colonize diverse environments [20]. *L. rossiae* DSM 15814^T^ was shown to grow well on modified Rogosa medium with XOS or its constituent pentose sugar xylose, or arabinose as the only carbon source. Growth of lactobacilli on XOS is not widely documented: only *Lactobacillusfermentum* (syn. *L. cellobiosus*) [32] and *Lactobacillus acidophilus* [33] have been reported to exhibit moderate growth on XOS, though this less vigorous compared to the growth capacity on this sugar shown by certain *Bifidobacterium* spp. [5, 34]. Furthermore, a gut-derived strain of *Lactobacillusparacasei* was shown to be positively stimulated by XOS [35].

*L. rossiae* DSM 15814^T^ is predicted to specify a number of extracellular or cell wall-associated polysaccharide-degrading enzymes, as well as putative enzymatic pathways for the metabolism of arabinose and xylose-containing poly- and/or oligosaccharides [20]. In this study a β-d-xylosidase was identified in *L. rossiae* DSM 15814^T^ and successfully expressed in *Lc. lactis* subsp. *cremoris* NZ9000. β-d-xylosidase represents one of the key xylanolytic enzymes in supplying carbon and energy to a variety of organisms [36, 37]. Sequential hydrolysis of xylans leads to the release of xylose, which is then transformed to the common metabolic intermediate xylulose 5-phosphate [38]. In *L. rossiae* DSM 15814^T^ the genes involved in the xylose and xylo-oligosaccharides utilization pathway appear to be clustered in a single operon designated here as *xyl.* The deduced amino acid sequence of the LROS_1108 gene belonging to the *xyl* cluster and designated here as *xylA*, showed high similarity to (predicted) β-d-xylosidases encoded by various lactic acid bacteria, a glycosyl hydrolase family 43 enzyme [14]. Recombinantly produced XylA was shown to catalyse the complete hydrolysis of XOS to xylose. The presence of a highly specific xylosidase is expected to play a crucial role in facilitating the utilization of XOS. As shown by transcriptome analysis, all *xyl* cluster genes were variously up-regulated when *L. rossiae* DSM 15814^T^ was grown in the presence of XOS and D-xylose, compared to cells grown when maltose was the sole carbon source. Furthermore, the up-regulation of the *ara* gene cluster, predicted to encode enzymes for l-arabinose utilization, was observed when, as expected, this sugar was present as the sole carbon source. Although these findings provide insights into the genes required for growth on XOS, our data are insufficient to reconstruct the xylanolytic machinery of *L. rossiae* DSM 15814^T^. Nonetheless, β-xylosidase is generally associated with other xylanolytic enzymes in order to impart an efficient conversion of xylo-oligosaccharides into xylose [36], and the involvement of other, perhaps secreted xylosidases cannot be ruled out.

Many bacterial β-d-xylosidases represent large dimeric or trimeric proteins with molecular masses in excess of 100 kDa [9, 39]. Monomeric β-d-xylosidases with a molecular mass less than 100 kDa have also been described [40]. The apparent molecular weight of the β-d-xylosidases of *L. rossiae* is higher than those encoded by *Bifidobacterium breve* K110 (49 kDa) [40] and *Clostridium cellulolyticum* (43 kDa) [41], and comparable to that of *Lactobacillus brevis* NCDC01 (58 kDa) [9].

The β-xylosidase of *L. rossiae* DSM 15814^T^ was shown to exhibit pH and temperature optima of ca. 6.0 and 40 °C, respectively. It was highly active (>80 %) in the range of pH of 5.0–7.0. β-xylosidases from other bacteria are usually active and stable at neutral pH [42] and a temperature range of 35–45 °C. The β-xylosidase purified from *L. brevis* NCDC01 was shown to exhibit pH and temperature optima of 6.0 and 40 °C [9], while the β-xylosidases of *Bacillus thermantarcticus* [43] and *Bifidobacterium adolescentis* were characterized by optimal activity at 5.0–7.0 and 50–70 °C [44]. Based on enzyme properties, the β-xylosidase of *L. rossiae* was found to be similar to that purified from *L. brevis*. *L. rossiae* is a novel species [23] that is able to adapt to different environments [21, 23–25, 28], and therefore further insights are desirable to exploit its biotechnological features and understand its metabolic versatility.

β-xylosidase activity appears to be produced by *L. rossiae* DSM 15814^T^ during sourdough fermentation, even though at a level slightly lower than when the strain is cultivated in WFH.

Cocktails of xylanases including β-d-xylosidase are used to hydrolyze glycosidic linkages from xylan and/or arabinoxylan liberating XOS in a first step, which are subsequently degraded releasing smaller water-soluble fragments, positively correlated with bread properties such as oven spring and volume [45]. Although *L. rossiae* was shown to be incapable of utilizing xylan and/or arabinoxylan, its β-xylosidase activity might contribute to the subsequent degradation of the XOS released by other xylanases.

## Conclusions

The activity of the β-d-xylosidase encoded by *L. rossiae* DSM 15814^T^ allows this bacterium to effectively fulfil the role of a xylo-oligosaccharide (XOS)-metabolizing cell factory. This study highlights some biochemical traits of *L. rossiae* that may be exploited for biotechnological purposes such as its use as a starter for the sourdough process or as a potential probiotic. The XOS metabolic trait has no doubt implications for the environmental adaptation by *L. rossiae* DSM 15814^T^, and functional genomic studies are underway to better understand XOS metabolism by *L. rossiae*.

## Methods

### Bacterial strains, plasmids, media and growth conditions

*L. rossiae* DSM15814^T^, previously isolated from sourdough, was routinely cultivated at 30 °C for 24 h on Sourdough Bacteria Medium (SDB). To assay growth of this strain on xylo-oligosaccharides (XOS) (Shandong Longlive Bio-Technology Co., China), xylan (Sigma-Aldrich, Ireland), d-xylose or l-arabinose (Oxoid, Basingstoke, Hampshire, United Kingdom), xylan, rye arabinoxylan, wheat arabinoxylan, arabinan, arabinogalactan, xyloglucan, as the sole carbon source, an *L. rossiae* DSM15814^T^ culture grown on SDB broth for 24 h at 30 °C was harvested by centrifugation (10,000×*g*; 10 min at 4 °C), washed twice in 50 mM sterile potassium phosphate buffer (pH 7.0), resuspended in sterile distilled water to a final optical density at 620 nm (OD_620_) of 2.5 (final cell number corresponding to ca. log 9.0 CFU ml^−1^), and then used to inoculate (2 %, [vol/vol]; initial cell number corresponding to ca. log 7.0 CFU ml^−1^) the modified Rogosa medium containing 0.5 % (wt/vol) of each of the above mentioned carbohydrate. Modified Rogosa medium containing the same amount [0.5 % (wt/vol)] of maltose was used as control.

Fermentations were carried out at 30 °C for 24 h. Growth kinetic data was determined by plating on SDB agar, and data were modeled according to the Gompertz equation as modified by Zwietering et al. [46]: y = k + A exp{−exp[(μ_max_ e/A)(λ−t) + 1]}, where k is the initial level of the dependent variable to be modeled (log CFU ml^−1^ h^−1^), A is the difference in cell density between inoculation and the stationary phase, μ_max_ is the maximum growth rate (expressed as Δ log CFU ml^−1^ h^−1^), λ is the length of the lag phase (expressed in hours), and t is the time. The experimental data was modeled through the non-linear regression procedure of the statistics package Statistic for Windows (Statsoft, Tulsa, Oklahoma, USA).

*Lactococcus lactis* subsp. *cremoris* NZ9000 [47] was cultivated at 30 °C for 24 h in M17 broth (Oxoid), which was supplemented with 0.5 % (wt/vol) d-glucose (GM17 broth). Recombinant *Lc. lactis* cells containing pNZ8048 or derivatives were selected on GM17 agar plates supplemented with 5 µg ml^−1^ of chloramphenicol.

### DNA manipulations and plasmid construction

Chromosomal DNA was isolated from *L.rossiae* DSM15814^T^ as previously described [48]. Plasmid DNA was isolated from *Escherichia coli* with the Roche High Pure plasmid isolation kit (Roche Diagnostics, Basel, Switzerland). An initial lysis step was performed with 30 mg ml^−1^ of lysozyme for 30 min at 37 °C prior to plasmid isolation from *E. coli*. The single-stranded oligonucleotide primers used in this study were synthesized by Eurofins (Ebersberg, Germany) (Table 1). Gene functional annotations were made by BLAST. The primers used for cloning (1108CHisPstF/1108CHisXbaR) were designed so as to incorporate two different endonuclease enzyme sites (PstI and XbaI) in order to facilitate subsequent cloning in the expression vector pNZ8048 and an in-frame His6-encoding sequence into reverse primer 1108CHisXbaR to facilitate downstream protein purification. Standard PCRs were performed with Taq master mix (Qiagen GmbH, Hilden, Germany), while high-fidelity hot start KOD DNA polymerase (Millipore, Ireland) was used to generate a DNA fragment, encompassing the predicted β-d-xylosidase-encoding gene, *xylA*. *L.rossiae* colony PCRs were carried out according to O’Connell Motherway et al. [49]. PCR fragments were purified employing the Roche High Pure PCR purification kit (Roche Diagnostics). Introduction of plasmid DNA into competent *Lc. lactis* by electroporation was carried out as previously reported [50]. The fragment encompassing *xylA* and the nisin-inducible translational fusion plasmid pNZ8048 were both digested with PstI and XbaI, and then ligated [51]. The ligation mixtures were introduced into *Lc. lactis* subsp. *cremoris* NZ9000 by electroporation, and transformants were selected based on chloramphenicol resistance. The plasmid contents of a number of chloramphenicol-resistant transformants were screened by restriction analysis, and the integrity of the plasmid, designated here as pNZ8048-*xylA*, retrieved from positively identified clones was verified by sequencing (MWG Biotech AG, Ebersberg, Germany).

**Table 1:** Oligonucleotide primers used in this study

Primer	Sequence (5′–3′)	Comments
Xyl.a (F)	CGCGGCTAAGATAGGTTCC	Hypotetical protein/Aldoese 1-epimerase (EC 5.1.3.3) forward
Xyl.a (R)	CTGTCGTGGTCAACGTGTTC	Hypotetical protein/Aldoese 1-epimerase (EC 5.1.3.3) reverse
Xyl.b (F)	GGAGAACTCGCATGACAATG	β-d-xylosidase (EC 3.2.1.37)/Xyloside transporter (*XynT*) forward
Xyl.b (R)	GGTTGTTCATAGCCAGCATAATC	β-d-xylosidase (EC 3.2.1.37)/Xyloside transporter (*XynT*) reverse
Xyl.c (F)	GTTGTGTCAGTGGCTGCTG	d-xylose proton symporter (*XylT*)/Xylose isomerase (EC 5.3.1.5) forward
Xyl.c (R)	GTGTCAACGATGTAGTGGTTG	d-xylose proton symporter (*XylT*)/Xylose isomerase (EC 5.3.1.5) reverse
Xyl.d (F)	GAGTTATGTATTGGGTGTGGAC	Xylulose kinase (EC 2.7.1.17)/Transcriptional regulator forward
Xyl.d (R)	GAACGCGATGCGTAATAAGAG	Xylulose kinase (EC 2.7.1.17)/Transcriptional regulator reverse
1108CHisPstF	aaaaaaCTGCAGatgaaaattcaaaatcctgtactg	Restriction site (PstI) flanked by homologous sequence LROS_1108, forward
1108CHisXbaR	aaaaaaTCTAGACATCACCATCACCATCACttattttgtttctggcaattctttg	Restriction site (XbaI) flanked by 6 x His tag and homologous sequence LROS_1108, reverse
araRS (F)	GTCTAATGAATCCCTGCTG	Transcriptional regulator ArsR family reverse
araRS (R)	CCAAAAATCGTGCAGCCG	Transcriptional regulator ArsR family forward
araR (R)	AACAGTAGCATCAGCAGGTT	Transcriptional repressor 2C Gnt family (*araR*) reverse
araR (F)	AGTAAAATGATTGGCGTCAT	Transcriptional repressor 2C Gnt family (*araR*) forward
araB (R)	GTAATTGGCGTATTCAAAGC	Ribulokinase (*araB*) reverse
araB (F)	CTAGAACAGGTTTGGACTGG	Ribulokinase (*araB*) forward
araD (R)	GACTTCGCATTATTTTGACC	l-ribulose-5-phosphate-4-epimerase (*araD*) reverse
araD (F)	GAAAAGGGGTTATTCGTCAT	l-ribulose-5-phosphate-4-epimerase (*araD*) forward
araA (R)	CGTTGATTTGTTCTTCGTCC	l-arabinose isomerase (*araA*) reverse
araA (F)	GTTAGCAGTTCCAGATTACG	l-arabinose isomerase (*araA*) forward

The β-D-xylosidase sequence of XylA from *L. rossiae* DSM 15814^T^, as well as deduced β-d-xylosidase sequences from lactic acid bacteria belonging to *Lactobacillus*, *Leuconostoc*, *Weissella* and *Lactococcus* genera (GenBank database), were used for comparative analysis. Sequence alignments were carried out using the MultiAlign program and Clustal W [52]. Phylogenetic tree construction was performed using the neighbor-joining program from the Clustal X software (National Center for Biotechnology Information) and visualized with the TreeView program.

### Heterologous XylA production

Nisin-inducible gene expression was performed as described previously [53]. When a gene is placed downstream of the inducible promoter P_nisA_ of plasmid pNZ8048 [47], transcription of that gene will be induced following the addition of sub-inhibitory concentrations of nisin [54]. In brief, 400 ml of GM17 broth supplemented with 200 μl of chloramphenicol (10 mg ml^−1^) was inoculated with a 2 % inoculum of *Lc. lactis* strains harboring pNZ8048.*xylA*, followed by incubation at 30 °C until an OD_600_ of 0.2 was reached. At this point, protein expression was induced by the addition of nisin (Nisaplin™, DuPont; 40 ng ml^−1^), followed by continued incubation for a further 4 h. Un-induced control was incubated as above without the addition of Nisaplin™. Cells were harvested by centrifugation (7000×*g*, 10 min), resuspended into buffer (50 mM Tris–HCl pH 8, 300 mM NaCl and 20 mM CaCl_2_), and stored at −20 °C overnight until further use. The thawed cell suspension was incubated in lysis buffer 50 mM Tris–HCl pH 8, 300 mM NaCl, 50 mM CaCl_2_ and 25 mg ml^−1^ lysozyme (Sigma-Aldrich, Ireland) at room temperature for 30 min, then sonicated in an ice bath at 200 W for 2.5 min (alternating between 30 s of sonication and 10 s cooling), and centrifuged (14,000×*g* for 25 min at 4 °C). The supernatant, containing the soluble protein fraction, and pellet (insoluble protein fraction and cell debris) were stored at 4 °C until further analysis. Aforementioned fractions were analyzed by SDS–polyacrylamide gel electrophoresis, followed by fixation and staining with Coomassie brilliant blue R-250. The apparent molecular weight of the protein was estimated by comparison with rainbow-pre-stained, low-molecular-weight protein markers (New England Biolabs, Herefordshire, United Kingdom). Protein concentration was determined by the Bradford method [55]. Protein purification of the crude cell extract (CCE) from Nisaplin-induced *Lc. lactis* cells containing pNZ8048-*xylA* was performed using Ni–NTA matrices according to the manufacturers’ instructions (Qiagen).

### Assessment of XOS hydrolysis by means of high-performance anion exchange chromatography- pulsed amperometric detection (HPAEC-PAD)

To assess the β-d-xylosidase activity of XylA, 50 μl of purified XylA protein or CCE (6.27 mg ml^−1^) was incubated at 37 °C for 24 h with 0.5 % (wt/vol) XOS in 0.1 M 3-(N-morpholino) propanesulfonic acid 4-morpholinepropanesulfonic acid (MOPS) buffer (pH 7.0). The reaction was stopped by heat treatment at 99 °C for 2 min and the mixture was centrifuged at 12,000×*g* for 5 min. The hydrolysis of XOS was assessed by HPAEC-PAD analysis employing a Dionex ICS-3000 system (Dionex, Sunnyvale, CA, USA) as described by Watson et al. [56]. The end-product d-xylose was identified by comparison with the retention time of the standard (5 min).

### β-d-xylosidase assay under in vitro sourdough conditions

The enzyme properties of His-tagged XylA were determined by assaying the amount of *p*-nitrophenol (*p*NP) released from *p*-nitrophenyl-β-d-xylopyranoside (*p*NPX) substrate at 37.5 ± 1 °C, as described by Lasrado and Gudipati [9]. The reaction mixture, consisting of 5 mM *p*NPX in 50 mM phosphate buffer (900 µl, pH 5.7), was incubated with 50 µl (6.27 mg ml^−1^) XylA-containing CCE for 10 min. The reaction was stopped by the addition of 100 µl of saturated solution of sodium tetraborate and the amount of *p*NP released was determined by measuring the absorbance at 410 nm. Since *L. rossiae* was isolated from sourdough, enzyme activity was also assayed in sourdough and in WFH as follows. First, 300 g of dough (DY, 160) was prepared with 187.5 g of flour and 112.5 g of water, containing *L. rossiae* cells at a final cell density of approximately log 7 CFU g^−1^. The dough was supplemented with *p*NPX at a final concentration of 5 mM and incubated at 30 °C for 24 h. The amount of *p*NP released was determined by measuring the absorbance at 410 nm prior and after the incubation. The *p*NP was extracted using phosphate buffer (pH 6.0). Four milliliters of buffer was added to 1.6 g of dough, homogenized for 180 s and centrifuged for 10 min at 10,000×*g* at 4 °C. The activity was expressed as the increase of absorbance per min per mg of protein. WFH was prepared as described by Gobbetti [57]. The medium was inoculated with *L. rossiae* at a final cell density of approximately log 7 CFU ml^−1^. After 24 h of fermentation at 30 °C, the CCE was obtained as described for Nisaplin-induced cells and the enzyme activity was determined as described by Lasrado and Gudipati [9].

### Effect of pH and temperature on β-d-xylosidase activity

The optimum pH for the XylA-mediated β-d-xylosidase activity was determined by incubating 50 μl CCE (6.2 mg ml^−1^) from Nisaplin™-induced *Lc. lactis* NZ9000 cells containing plasmid pNZ8048-*xylA* at 37 °C for 10 min with *p*NPX. The different values of pH were obtained using 0.05 M Na-acetate (3.0–6.0), phosphate (6.0–7.0) and Tris–HCl (7.0–9.0) buffer. The optimum temperature for XylA activity was determined at pH 6.0 (50 mM phosphate buffer), in the range of 20–70 °C using *p*NPX as a substrate (see previous paragraph for a detailed description of the employed assay).

### RNA-seq analysis and phylogenetic study

Approximately 10 μg of total RNA was obtained from *L. rossiae* DSM15814^T^ cells grown in modified Rogosa medium, containing XOS, d-xylose, l-arabinose or maltose as the only carbon source. Ribosomal RNA was removed using the Ribo-Zero rRNA removal kit according to supplier’s instructions (Epicentre, Madison, WI, USA). The rRNA depletion step was verified by a 2200 TapeStation (Agilent technologies, USA). Then, 100 ng of rRNA-depleted RNA was fragmented using RNaseIII (Life Technologies, USA) followed by size evaluation using a 2200 TapeStation (Agilent technologies, USA). A whole transcriptome library was constructed using the Ion Total-RNA Seq Kit v2 (Life Technologies, USA). Barcoded libraries were quantified by qRT-PCR and each library template was amplified on Ion Sphere Particles using Ion One Touch 200 Template Kit v2 (Life Technologies, USA). Samples were loaded into 316 Chips and sequenced on the PGM (Life Technologies, USA). Sequencing reads were depleted of adapters, quality filtered (with overall quality, quality window and length filters) and aligned to the *L. rossiae* DSM 15814^T^ reference genome through BWA [58]. The sequence data have been submitted to the Sequence Read Archive database of the National Center for Biotechnology Information under accession no. SRP072553. Counts of reads that corresponded to ORFs were performed using HTSeq (http://www-huber.embl.de/users/anders/HTSeq/doc/overview.html) and analysis of the count data was performed using the R package DESeq [59].

### Nucleotide sequence analysis

Sequence data were obtained from the Artemis-mediated genome annotations of *L. rossiae* DSM15814^T^ [20]. Database searches were performed using the non-redundant sequence database accessible at the National Center for Biotechnology Information website (http://www.ncbi.nlm.nih.gov) with BLAST [60]. Sequence analysis was performed employing Seqbuilder and Seqman programs of the DNASTAR software package (DNASTAR, Madison, WI).

## Additional file

10.1186/s12934-016-0473-z Effect of pH (A) and temperature (B) on the β-xylosidase activity of *Lactobacillus rossiae* DSM 15814^T^; **Table S1**. Gene sequences BLAST alignment.
