# Supplementary material for: Cloning, expression and characterization of a β-d-xylosidase from Lactobacillus rossiae DSM 15814T
Source: Microb Cell Fact. 2016 May 3;15:72. doi: 10.1186/s12934-016-0473-z (PMC4855831; doi:10.1186/s12934-016-0473-z)
Supplement: Supplementary file 1 — 10.1186/s12934-016-0473-z Effect of pH (A) and temperature (B) on the β-xylosidase activity of Lactobacillus rossiae DSM 15814T; Table S1. Gene sequences BLAST alignment. [file 12934_2016_473_MOESM1_ESM.pdf]

Figure S1. Effect of pH (A) and temperature (B) on the β-xylosidase activity of *Lactobacillus rossiae* DSM 15814^{T}. Effect of pH was determined in Na-acetate (3.0 – 6.0), phosphate (6.0 – 7.0) and Tris-HCl (7.0 – 9.0) buffers, whereas the temperature was assayed in phosphate buffer (pH 6). The U refers to the increase of the absorbance at 410 nm in one minute per mg of protein. Reaction time 10 minutes.

(A)

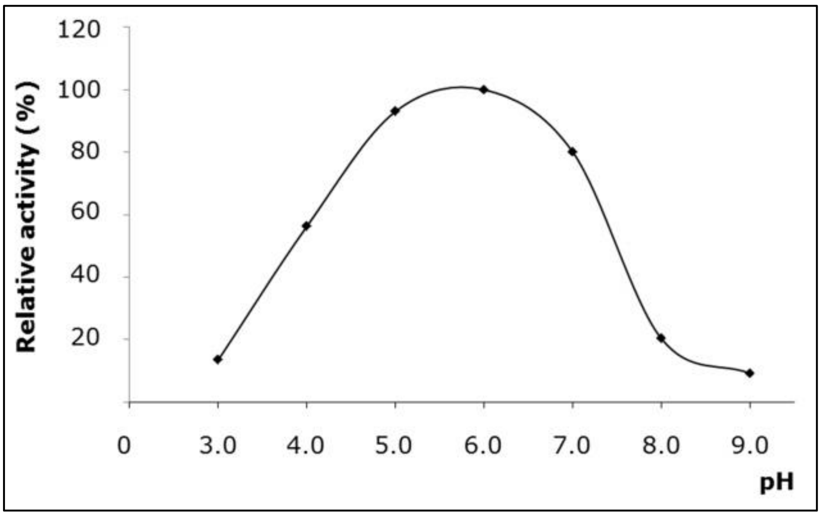

(B)

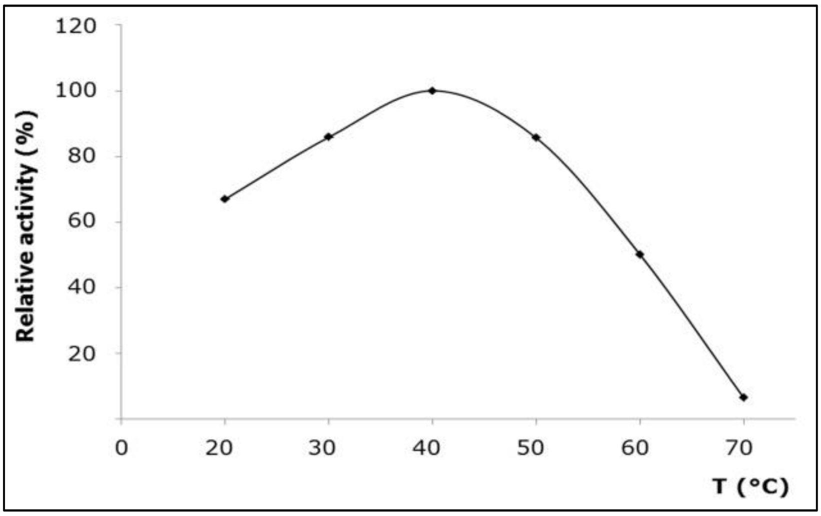

TABLE S1. Gene sequences BLAST alignment

Gene	Function	Accession number	E-value	Identity
		***xyl* cluster**		
**LROS_1106**	Hypothetical protein	121447	0.0	100%
**LROS_1107**	Aldose 1 epimerase	206431	0.0	100%
xylA	B-xylosidase	141219	0.0	99%
xynT	Xyloside transporter	99065	0.0	99%
xylT	D-xylose proton symporter	19897	3e-173	100%
xylI	Xylose isomerase	229077	0.0	100%
xylK	Xylulose kinase	25965	0.0	99%
xylR	Transcriptional regulator	190937	0.0	99%
		***ara* cluster**		
araA	L-arabinose isomerase	167475	0.0	100%
araB	Ribulokinase	240627	0.0	100%
araD	L-ribulose-5-phosphate-4-epimerase	53991	0.0	100%
araR	Transcriptional repressor 2C GnT family	116651	0.0	99%
araRS	Transcriptional regulator ArsR family	60305	0.0	99%
